# Engineering Microbial Cells for the Biosynthesis of Natural Compounds of Pharmaceutical Significance

**DOI:** 10.1155/2013/780145

**Published:** 2013-04-27

**Authors:** Philippe Jeandet, Yann Vasserot, Thomas Chastang, Eric Courot

**Affiliations:** ^1^Laboratory of Enology and Applied Chemistry, Research Unit on Vines and Wines in Champagne, UPRES EA 4707, Faculty of Sciences, University of Reims, P.O. Box 1039, 51687 Reims Cedex 02, France; ^2^Laboratory of Stress, Defenses, and Plant Reproduction, Research Unit on Vines and Wines in Champagne, UPRES EA 4707, Faculty of Sciences, University of Reims, P.O. Box 1039, 51687 Reims Cedex 02, France

## Abstract

Microbes constitute important platforms for the biosynthesis of numerous molecules of pharmaceutical interest such as antitumor, anticancer, antiviral, antihypertensive, antiparasitic, antioxidant, immunological agents, and antibiotics as well as hormones, belonging to various chemical families, for instance, terpenoids, alkaloids, polyphenols, polyketides, amines, and proteins. Engineering microbial factories offers rich opportunities for the production of natural products that are too complex for cost-effective chemical synthesis and whose extraction from their originating plants needs the use of many solvents. Recent progresses that have been made since the millennium beginning with metabolic engineering of microorganisms for the biosynthesis of natural products of pharmaceutical significance will be reviewed.

## 1. Introduction

Genetic engineering of cells, particularly microorganisms, can be successfully applied for the development of strains dedicated to the overproduction of natural products. In fact, microbes are widely used for the biosynthesis of numerous valuable molecules such as antitumor, anticancer, antiviral, antiparasitic, antioxidant, immunological, agents, antibiotics and hormones as well as biofuels [1–7]. Biotechnology might represent a powerful means for large-scale synthesis of natural compounds as the use of microorganisms allows for low cost and rapid production of biologically active molecules through an environmentally benign route. Such approaches avoid organic solvents, heavy metal catalyzers, and strong acids and bases that are currently employed upon synthetic chemistry-based routes. Some natural compounds including nicotianamine for instance can reach unrealistic prices ($ 350,000 per gram). On the other hand, extraction of valuable natural products from native plant sources is difficult as these compounds generally accumulate in very small amounts, also needing the use of many solvents. As an example, the doses of taxol needed for the treatment of a single patient requires the sacrifice of two to four fully grown trees of *Taxus brevifolia*. Finally, microbial fermentations are very easy to transpose at the industrial level, as previously reported for the production of resveratrol [8].

Currently, *Escherichia coli*, on one hand, and *Saccharomyces cerevisiae*, on the other hand, are employed for the microbial synthesis of almost all natural products of interest, although new platform microorganisms are emerging [6].

This review summarizes, without being exhaustive, efforts that have been made since the millennium beginning with the metabolic engineering of microbes for the biosynthesis of natural products with a particular emphasis on compounds showing antibiotic, antitumoral, antiparasitic, antihypertensive, immunological, hormonal, and antioxidant activities and belonging to various chemical families, including terpenoids, polyphenols, polyketides, alkaloids, stilbenoids, amines, and proteins. 

## 2. Antibiotics

**Figure 1:**
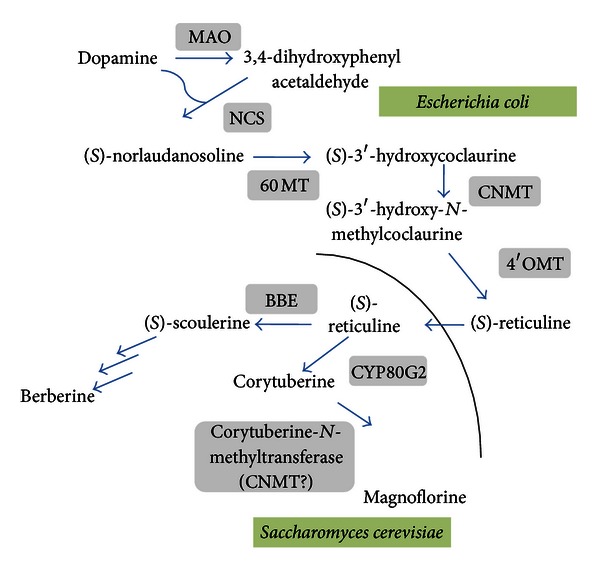
Benzylisoquinoline alkaloid biosynthetic pathway reconstructed in *Escherichia coli* and *Saccharomyces cerevisiae*. For *in vivo* production of *(S)*-reticuline, transgenic *E. coli* expresses biosynthetic genes *MAO*, *NCS*, *6OMT*, *CNMT,* and 4′*OMT*. For *in vivo* production of *(S)*-scoulerine and magnoflorine from reticuline originating from engineered *E. coli*, transgenic *S. cerevisiae* expresses, respectively, the genes *BBE, CYP80G2* and *CNMT*. All genes are from *Coptis japonica* except the gene *MAO* which originates from *Micrococcus luteus*. MAO: monoamine oxidase; NCS: norcoclaurine synthase; 6OMT: norcoclaurine 6-*O*-methyltransferase; CNMT: coclaurine-*N*-methyltransferase; 4′OMT: 3′-hydroxy-*N*-methylcoclaurine-4′-*O*-methyltransferase; BBE: berberine bridge enzyme; CYP80G2: P450 corytuberine synthase.

 Among the alkaloid family which are nitrogen-containing molecules of low molecular weight synthesized by numerous plants, fungi, bacteria, and animals, benzylisoquinoline alkaloids constitute a remarkable group of pharmaceutical molecules including, for example, the narcotic analgesic morphine and the antibacterial agents berberine, scoulerine, palmatine, and magnoflorine. Besides, these alkaloids have been reported such as anti-human immunodeficiency virus (HIV) and antioxidant and anticancer agents (see [3, 4] and the references therein). Because these compounds are too complex for cost-effective chemical total synthesis, microbial systems for benzylisoquinoline-type alkaloid production were used [9, 10], but attempts to reconstruct the entire metabolic pathway leading to *(S)*-reticuline and, beyond this branch-point biochemical intermediate, to the synthesis of many other related alkaloids have been achieved recently by the group of Minami and coworkers [11]. They established a microbial system, combining two different microorganisms together with microbial and plant-derived genes. Reticuline production was achieved first in engineered *Escherichia coli* and the biosynthesis of specific alkaloids was terminated using a coculture of engineered* E. coli* and *Saccharomyces cerevisiae* [11]. This study resulted in (i) the biosynthesis of *(S)*-reticuline, the key intermediate in the formation of benzylisoquinoline alkaloids, only when using a methyl group donor and (ii), the production of specific alkaloids, magnoflorine, and corytuberine on one hand, and, on the other hand, scoulerine depending on the targeted genes. Reticuline in engineered *E. coli* was obtained as follows (Figure 1): dopamine was converted first to 3,4-dihydroxyphenylacetaldehyde by a monoamine oxidase from *Micrococcus luteus*; *(S)*-norlaudanosoline was synthesized by the condensation of dopamine and 3,4-dihydroxyphenylacetaldehyde by a norcoclaurine synthase from *Coptis japonica*, then leading to *(S)*-3′-hydroxycoclaurine via a norcoclaurine 6-*O*-methyltransferase from *C. japonica*. *(S)*-3′-hydroxy-*N*-methylcoclaurine was obtained through the action of coclaurine-*N*-methyltransferase from *C. japonica*, leading to *(S)*-reticuline via the 3′-hydroxy-*N*-methylcoclaurine-4′-*O*-methyltransferase from *C. Japonica*. When *S*-adenosyl-L-methionine (SAM) was used as a methyl group donor, the engineered biosynthesis pathway leads predominantly to the stereospecific *(S)-*reticuline with a yield of 55 mg/L within 1 h. *S. cerevisiae* expressing heterologous genes were added to *E. coli *cells engineered with the reticuline biosynthetic genes with 5 m*M* dopamine in the medium after a certain period of culture. *S. cerevisiae* was engineered to harbor the genes encoding the P450 enzyme, corytuberine synthase (CYP80G2), and a CNMT from *C. japonica* (possibly a CNMT-like methyltransferase), leading to magnoflorine at a yield of 7.2 mg/L within 72 h without any addition of SAM. Otherwise, the second alkaloid produced, *(S)*-scoulerine, was obtained with a *S. cerevisiae *strain expressing the berberine bridge enzyme (BBE) from *C. japonica* at a yield of 8.3 mg/L within 48 h. The conversion efficiencies of reticuline to magnoflorine or scoulerine in *S. cerevisiae* cells were 65.5% and 75.5%, respectively. This underlines that such a working system results in the efficient production of benzylisoquinoline alkaloids in engineered microbes. The system could be generalized and applied to the production of many other alkaloids of medical interest [11].

Recently, an important modification of the pathway leading to *(S)*-reticuline was reported by the same group [12]. An *E. coli* strain that over produces L-tyrosine by amino-acid fermentation was first generated. This was accomplished by three steps of genetic engineering. Production of dopamine was then obtained via the introduction in *E. coli* of a tyrosinase and its adaptor protein from *Ralstonia solanacearum*, able to convert L-tyrosine to L-dihydroxyphenylalanine (L-DOPA) along with a L-DOPA decarboxylase from *Pseudomonas putida*, leading to L-dopamine. This short pathway closed the gap between L-tyrosine and the synthetic pathway to reticuline via the monoamine oxidase from *M. luteus* and leading to the 3,4-dihydroxyphenylacetaldehyde (see the previous section). In the final step of the fermentative production of *(S)*-reticuline, the dopamine-producing pathway described above was combined with the synthetic pathway from dopamine to *(S)*-reticuline.

 Engineering microbes also offers great opportunities for the biosynthesis of plant polyketides of medical interest including antibiotics (erythromycin, rifamycin), anticancer drugs (tetracyclines, anthracyclines, and epothilones), antiparasitic agents (avermectin), cholesterol-lowering agents (lovastatin), and immunosuppressants (rapamycin) [13]. They are synthesized from acyl-CoA precursors by polyketide synthases (PKSs) [14], enzymes that are grouped in three different classes: (i) type I refers to large and multifunctional enzymes; (ii) type II refers dissociable complexes usually composed of monofunctional enzymes found in bacteria [15]; (iii) type III consists of homodimeric enzymes of relatively small size found in plants [16] as well as in bacteria [17] and fungi [18]. Type III PKSs catalyze iterative condensations of malonyl-CoA units with a great variety of CoA-linked starter substrates (see below).

**Figure 2:**
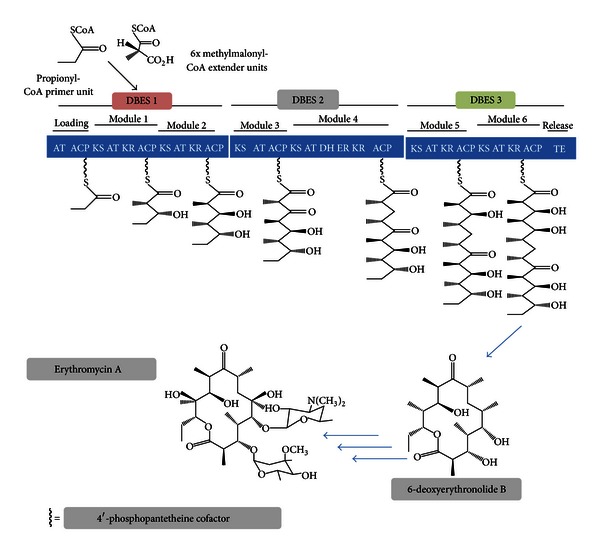
Biosynthesis of deoxyerythronolide B in the route to erythromycin A (adapted from [19]). Deoxyerythronolide B synthases (DEBS) catalyze iterative claisen-type condensations by progressively associating one propionyl-CoA primer unit with six methylmalonyl-CoA extender units. Each DEBS molecule contains two modules, these modules representing a physical location for a claisen-type condensation. Once loaded onto the ketosynthase (KS) of module 1 by the loading acyl transferase (AT) and the acyl carrier protein (ACP) domains, propionyl-CoA condenses with a methylmalonyl unit loaded onto module 1 ACP by the module 1 AT domain. After that, ketoreductases (KR), specific for each module, reduce the resulting ketone group. The chain then passes in a progressive manner from module 1 ACP (via a phosphopantetheine cofactor) to the KS domain of the next module (module 2). Following these iterative condensations, the polyketide chain grows before being released and cyclized by a thioesterase/claisen cyclase (TE). Other abbreviations: ER are enoyl reductase and DH: dehydratase.

The macrocyclic core of erythromycin, 6-deoxyerythronolide B (6-dEB), a broad spectrum antibiotic synthesized by the soil bacterium *Saccharopolyspora erythraea*, is a prototype of polyketides. As mentioned above, polyketides are obtained from single building blocks, acetyl-CoA, propionyl-CoA, and methylmalonyl-CoA (Figure 2) [19], requiring the production of the last two compounds reengineering of *E. coli* metabolism due to limited capabilities for molecular biological manipulation of microorganisms especially actinomycetes [20]. For an efficient 6-dEB production, the intracellular activity of the deoxyerythronolide synthase (DEBS) catalyzing formation of the macrocyclic core of erythromycin has to be well synchronized with precursor biosynthesis. 

 First attempts for engineering this polyketide pathway in *E. coli* addressed expression of the genes encoding DEBS. Each of the three genes encoding for DEBS 1, DEBS 2, and DEBS 3 from *S. erythraea *was cloned into *E. coli* BL21 (DE 3) along with the *sfp *phosphopantetheinyl transferase gene from *Bacillus subtilis*, the plasmid-based coexpression of this gene facilitating posttranslational modification of the DEBS protein in *E. coli *[20]. A single copy of the *sfp* gene under the control of the T7 RNA polymerase promoter was thus integrated in the *prp* operon of strain BL21 (DE 3), yielding *E. coli* strain BAP1. This site was chosen because it suppresses the possibility that the bacterium cell uses propionate, a starting block for 6-dEB biosynthesis, as a source for catabolism and anabolism. Secondly, along with the *sfp* gene, the *prpE* gene of the strain BAP1 was placed under control of an IPTG inducible T7 promoter. PrpE is thought to convert propionate into propionyl-CoA, thus allowing accumulation of this precursor inside the cell that is well synchronized with DEBS expression. Finally, a suitable pathway for the biosynthesis of *(2S)*-methylmalonyl-CoA was engineered in *E. coli*. The strain BAP1 was thus transformed with plasmids harboring the propionyl-CoA carboxylase genes *pccA* and *pccB* from *Streptomyces coelicolor*. Coexpression of the birA biotin ligase from the bacterium was shown to enhance the activity of the biotinylated subunit pccA [20].

To resume, engineering of *E. coli* to produce 6-dEB included introduction of the DEBS genes from *S. erythraea*, introduction of a propionyl-CoA carboxylase from *B. subtilis*, introduction of a propionyl-CoA carboxylase from S. *coelicolor *for *(2S)*-methylmalonyl-CoA biosynthesis and deletion of the endogenous *prpRBCD* genes to avoid utilization of propionate as a carbon source for catabolism and anabolism of the cell along with overexpression of the endogenous *prpE* and *birA* genes (to ensure conversion of propionate to propionyl-CoA and to enhance conversion of propionyl-CoA to *(2S)*-methylmalonyl-CoA, resp.). A major challenge in metabolic engineering is indeed to achieve an optimal flow through a given heterologous metabolic pathway for high yield and productivity. The best performing system led to a calculated specific productivity of 0.1 mmol, that is, 38.6 mg of 6-dEB/g cellular protein/day.

Production of 6-dEB in *E. coli* was improved further by the same group [21] by cloning an *S*-adenosylmethionine (SAM) synthase gene (*metK*) from *Streptomyces spectabilis *into an expression plasmid under the control of an inducible T7 promoter according to the assumption that the production of several antibiotics (for instance, streptomycin and erythromycin) is improved by increasing the intracellular concentration of SAM. The same observation was made with the production of nicotianamine by Wada and coworkers [22], indicating that increased SAM levels may be a generally useful tool for improving the production of various natural products. This plasmid was introduced into the engineered BAP1 engineered *E. coli* strain described above [20], improving the specific productivity of 6-dEB from 10.86 mg/L/OD_600_ to 20.08 mg/L/OD_600_ without or with heterologous *metK* expression within 5 days [21].

## 3. Antitumor Antibiotics

 Important human antibiotics and antitumor antibiotics such as tetracyclines and anthracyclines are of bacterial origin. These compounds are synthesized by bacterial type II polyketide synthases (PKSs). The main limitation for reconstituting the biosynthesis of aromatic polyketides in *E. coli *consists in the inability to generate the elongated poly-*β*-ketone backbone from malonyl-CoA, the starting block of the synthesis pathway [24]. This requires a minimal PKS comprising a ketosynthase- (KS-) chain length factor (CLF) and an acyl-carrier protein (ACP). In fungi, PKSs are megasynthases in which, unlike bacterial aromatic PKSs, the enzymatic components are not dissociated. Megasynthases include a starter-unit acyltransferase (SAT), a KS, a malonyl-CoA:acyl carrier protein (ACP) transacylase (MAT), a product template (PT), an acyl-carrier protein (ACP), and a thioesterase/claisen-cyclase (TE/CLC) (Figure 2) (see [24] and the references therein). A fungal megasynthase, the PKS4 from *Gibberella fujikuroi*, can be expressed by E.coli and the biosynthesis of aromatic polyketides can aslo be reconstituted in *E. coli *[25]. In order to produce polyketides with cyclization regioselectivities not observed among fungal polyketides, the PKS4 KS_MAT didomain and the ACP domain were extracted as stand-alone proteins, leading to a dissociated PKS4 minimal PKS. Such an approach mimics the dissociation of the components that are observed in bacterial type II minimal polyketide synthases [24]. In a second approach, Tang and coworkers [24] used a compact synthetic polyketide megasynthase PKS_WJ consisting of KS, MAT, and ACP on a single polypeptide. Two plasmids encoding the PKS_WJ were transformed into the *E. coli* strain BAP1. Under fed-batch fermentation, high cell density cultures of engineered *E. coli* lead to the production of 3 mg/L of the aromatic polyketide anthraquinone within 60 h after addition of isopropyl *β*-D-1-thiogalactopyranoside. In contrast, no polyketide production was observed in the dissociated PKS4 minimal PKS. Using engineered fungal PKSs in bacteria thus paves the way for a more general approach towards the production of various aromatic polyketides.

## 4. Anticancer Drugs

**Figure 3:**
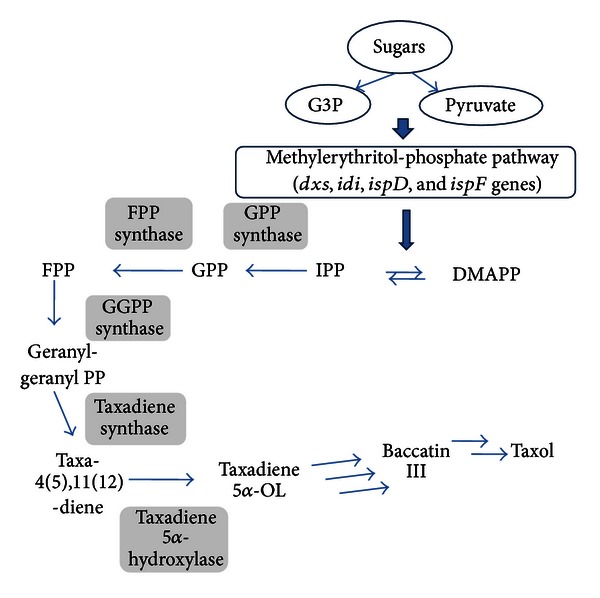
Pathway for *in vivo* production of taxadiene and 5-*α*-hydroxytaxadiene in the route to taxol via the methylerythritol phosphate pathway in *E. coli *(adapted from [23]). Genes *dxs*, *idi*, *ispD,* and *ispF*, respectively, encoding for 1-deoxy-D-xylulose-5-phosphate synthase, isopentenyl diphosphate isomerase, isoprenoid synthase domain, and 2C-methyl-D-erythritol-2,4-cyclodiphosphate synthase. IPP: isopentenyl diphosphate; DMAPP: dimethylallyl diphosphate; GPP: geranyl diphosphate; FPP: farnesyl diphosphate; GGPP: geranylgeranyl diphosphate.

Taxol (paclitaxel) and its structural analogs are potent and commercially successful anticancer drugs [26, 27]. Taxol is a diterpene originating from the isoprenoid (terpenoid) pathway. Opportunities for biosynthesis of natural terpenoid drugs using engineered microorganisms have been reviewed elsewhere [28–30]. Terpenoids are synthesized from the condensation of two C_5_ units, respectively, isopentenyl-pyrophosphate (IPP) and its isomer dimethylallyl-pyrophosphate (DMAPP), as starting blocks, both originating from either the mevalonate pathway or the 2C-methyl-D-erythritol-4-phosphate pathway (Figure 3) [31–35]. Condensation of these two C_5_ units and larger IPP- and DMAPP-derived building blocks such as the C_10_ unit, geranyl pyrophosphate (GPP), the C_15_ unit, farnesyl pyrophosphate (FPP), and the C_20_ unit, geranylgeranylpyrophosphate (GGPP), yields monoterpenes, sesquiterpenes (for example, artemisin, see below), triterpenes (steroids), tetraterpenes (carotenoids), and diterpenes such as taxol.

Taxol has been isolated from *Taxus brevifolia* (Pacific yew tree) (see [23] and the references therein). Unfortunately, sufficient taxol dosage for one patient requires sacrificing two to four fully grown trees of this species. Moreover, the chemical synthesis of this compound is extremely complex (requiring 35 to 51 steps), with a highest total yield of the best synthesis of 0.4% (see [23] and the references therein). Although a semisynthetic route using baccatin III (an intermediate in the taxol biosynthetic pathway isolated from plant sources) (Figure 3) was described, limitations were encountered in terms of productivity and scalability (see [23] and the references therein). Metabolic engineering of microbes thus appears along with plant cell cultures, as a valuable alternative for the production of such compounds, which eliminates reliance on yew tree plantations. As the biosynthesis of taxol comprises many enzyme-catalyzed steps, research was limited to attempts made towards the production of taxadiene, the first committed taxol intermediate. Metabolic engineering of taxadiene production as an essential step towards taxol production was first described in yeast *S. cerevisiae *[38]. In the best producing system, heterologous expression of the *Sulfolobus acidocaldarius *geranylgeranyl-diphosphate synthase along with the codon-optimized taxadiene synthase from *Taxus chinensis*, a truncated version of yeast 3-hydroxyl-3-methylglutaryl-CoA reductase (tHMG-CoA reductase) (which was also shown to increase production of artemisinic acid in recombinant yeast, see the next section and [36]) and the *UPC2-1* transcription factor gene, leads to taxadiene levels of 8.7 mg/L [38].

To go farther in the production of taxol, Stephanopoulos and co-workers designed a multivariate modular approach and succeeded in increasing the titer of taxadiene up to 1 g/L [23]. The multivariate modular pathway engineering was composed of two modules separated at the level of isopentenyl pyrophosphate (IPP)/dimethylallyl pyrophosphate (DMAPP). The first module comprised an eight-gene, upstream, methylerythritol-phosphate (MEP) pathway native to *E. coli*, the pathway in which the expression of only four genes (*dxs*, *idi*, *ispD, *and *ispF*) that deemed to be rate-limiting was modulated. To channel the overflow flux from the starting blocks, IPP and DMAPP, towards taxol production, a second module comprised a two-gene (encoding for the geranyl geranyldiphosphate synthase GGPPS and the taxadiene synthase, TS, resp.) downstream, heterologous pathway to taxadiene. Using this modular approach, taxadiene production exhibited a 15,000-fold increase over the control, yielding 1.02 g/L in defined media with controlled glycerol feeding. This has to be compared with the milligrams of taxadiene obtained in previous studies [38].

As the cyclic olefin taxadiene undergoes multiple steps of stereospecific oxidations (including oxygenation at seven different positions mediated by CYP450-dependent monooxygenases) [39], acylations, and benzoylation to form baccatin III, the next step in taxol biosynthesis leading to taxadien-5*α*-ol was engineered. The first oxygenation step is the hydroxylation of the C5 position catalyzed by a CYP450, taxadien-5*α*-hydroxylase (Figure 3) [40]. To succeed in the functional expression of plant CYP450 in *E. coli*, engineering taxol P450 oxidation chemistry in the bacterium was performed by the construction of a chimera protein from taxadien-5*α*-hydroxylase with its redox partner, *Taxus* cytochrome P450 reductase. One of the chimera enzymes generated, At24T5*α*OH-tTCPR, carried out the first hydroxylation step with a yield higher than 98%, leading to taxadien-5*α*-ol along with a byproduct.

Owing to the fact that there are still six more hydroxylation steps (and probably some other modifications of the taxane core structure that have not yet been identified) until reaching the suitable taxol precursor, baccatin III, a lot of work remains for engineering the complete pathway to taxol in microbes.

The epothilones are potential anticancer agents useful particularly in case of paclitaxel- (taxol-) resistant tumor cell lines [41]. Epothilones A and B are synthesized by the action of a hybrid nonribosomal peptide synthetase (NRPS)/type I polyketide synthase (PKS) together with a P450 epoxidase that converts desoxyepothilone into epothilone, by the myxobacterium, *Sorangium cellulosum *[42]. Inactivation of the epoxidase or deletion of the gene encoding for this enzyme yields epothilones C and D as direct products of the NRPS/PKS. Heterologous expression of an entire epothilone gene cluster composed of six open reading frames (ORFs), *epoA*, *epoB*, *epoC*, *epoD*, *epoE,* and *epoF* encoding a single NRPS module, eight PKS modules, and a C-terminal thioesterase, has already been described in *Streptomyces coelicolor *but with a low yield of epothilones [43] *and Myxococcus xanthus*, the growth of which is slow compared to *E. coli *[44]. In a more recent work [42], the genes encoding the entire cluster *epoA*, *epoB*, *epoC*, *epoD*, *epoE,* and *epoF* were redesigned and synthesized to allow for expression into two polypeptides with compatible module linkers. Splitting this large protein into two polypeptides was necessary to obtain its expression in a soluble form along with the lowering of the incubation temperature (15°C), co expression of molecular chaperones, and switching from a T7 promoter to an arabinose-inducible P_BAD_ promoter. The entire cluster was expressed in the modified *E. coli* K207-3, which is a derivative of BL21 (DE3), the so-called strain BAP1 already mentioned in the production of the macrocyclic core of erythromycin, 6-deoxyerythronolide B [20]. This, in turn, resulted in the complete biosynthesis of epothilones C and D in *E. coli* as clearly evidenced by the LC/MS/MS analysis of *E. coli* extracts. The design of the synthetic epothilone genes together with *E. coli* expression provides an excellent platform for the production of epothilone analogues, according to protein engineering strategies, to redesign a large gene cluster.

## 5. Antiparasitic Agents

**Figure 4:**
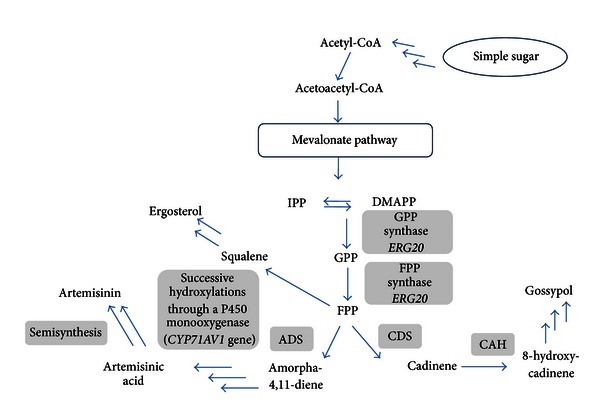
Engineering the pathways for the biosynthesis of artemisinic acid and 8-hdroxycadinene via the mevalonate pathway (adapted from [36, 37]). ADS: amorphadiene synthase; CDS: cadinene synthase; CAH: cadinene hydroxylase. For other abbreviations, see legend of Figure 3.

Terpenoids and their derivatives together with their roles in respiration, electron transport, photosynthesis, and hormone signaling may serve as antiparasitic agents. One of the most successful examples of terpenoid biosynthetic pathway engineering is the production of artemisinic acid, the precursor for the antimalarial agent artemisinin, in yeast [36]. Engineering of the pathway leading to artemisinic acid was achieved in yeast *S. cerevisiae* in three steps (Figure 4): (1) increasing farnesyl pyrophosphate (FPP) production, (2) conversion of FPP to amorpha-4,11-diene, and (3) three-step oxidation of amorphadiene to artemisinic acid. Combination, on one hand, of the overexpression of a truncated, soluble form of 3-hydroxy-3-methylglutaryl-CoA reductase (*tHMGR *gene) along with the down regulation of *ERG9* which encodes squalene synthase (leading to the production of sterols) increased amorphadiene production fivefold and twofold, respectively, in yeast. Downregulating *ERG9* and overexpressing *upc2-1* which enhances the activity of UPC2 (a global transcription factor regulating the biosynthesis of sterols in yeast) were combined with integration of an additional copy of *tHMGR *into the chromosome. Introducing the amorphadiene synthase gene from *Artemisia annua* to this high FPP engineered producer resulted in an increased production of amorphadiene up to 149 mg/L. Finally, overexpression of the gene encoding FPP synthase (*ERG20*) enhanced amorphadiene production by ca 10%, that is, 153 mg/L in the resulting engineered yeast strain EPY224 (about 500-fold the production previously reported [45]).

This transgenic yeast strain was transformed with a vector harboring a cytochrome P450 monooxygenase and its redox partner *CYP71AV1/CPR *from *A. annua* that performs a three-step oxidation of amorphadiene to artemisinic acid under the control of galactose-inducible promoters. The produced acid was efficiently transported out of the cell, making its purification relatively easy. In a one-litre aerated bioreactor, 115 mg of artemisinic acid was produced, of which 76 mg was recovered following a one-step silica gel column chromatographic separation of ether extracts from the washed buffers. Artemisinic acid is produced by the engineered *S. cerevisiae* strain EPY224 at a biomass fraction comparable to that produced by the plant but within few days for the yeast versus several months in *A. annua*. Artemisinin is then obtained from artemisinic acid by hemisynthesis [46]. Further experiments achieved by the same group have improved production of artemisinic acid in a transgenic yeast transformed with an amorphadiene synthase, an amorphadiene oxidase, and a cytochrome P450 reductase expressed from a single plasmid. Modulating the selection markers and using appropriate medium formulation, production of artemisinic acid in the engineered yeast reached 250 *μ*g/mL in shake-flask cultures and 1 g/L in bioreactors [47].

More recently, a novel semibiosynthetic route towards artemisinin has been reported using the long-chain fatty acid P450_BM3_ hydroxylase from *Bacillus megaterium* capable of forming artemisinic −11*S*, 12-epoxide from amorphadiene, this epoxide being an interesting intermediate to form dihydroartemisinic alcohol [48]. An engineered *E. coli* expressing this particular P450 hydroxylase yielded high titers of the epoxide (250 mg/L). Finally, engineering of the mevalonate pathway in Seo *E. coli* with equivalent genes from *Staphylococcus aureus* yielded an average amount of 22.7 g/L of amorphadiene in the culture medium [49].

Difficulties in efficient translations and functional expression of key enzymes including plant P450 enzymes are encountered in *E. coli*. To overcome this limitation, the production of functionalized terpenoids in *E. coli*, such as the conversion of cadinene to 8-hydroxycadinene, a precursor for the phytoalexin gossypol, was achieved by Chang and co-workers by cloning a cadinene hydroxylase along with a cytochrome P450 reductase (Figure 4) [37].

## 6. Antihypertensive Agents

Nicotianamine (NA) is an ubiquitous compound in plants thought to play a role, as a metal chelator, in the internal transport of metal nutrients as well as to be a precursor of mugineic acids secreted from the roots and facilitating iron solubilization in the soil. Interestingly, NA possesses antihypertensive effects via the inhibition of the angiotensin I-converting enzyme which catalyzes hydrolysis of angiotensin I to the potent vasoconstrictor angiotensin II [50]. NA biosynthesis is quite simple as it is obtained from the condensation of three *S*-adenosylmethionine (SAM) units by nicotianamine synthase (NAS). Large amounts of NA (766 *μ*g/g wet weight) were obtained following the introduction of the *Arabidopsis AtNAS2* gene into the yeast *Saccharomyces cerevisiae* strain SCY4 [22]. Two vectors for NAS expression in *S. cerevisiae* were constructed, the first one containing an integrative vector, pINNAS (strain SCY4-pINNAS) and the second one a high-copy episomal vector, pEPNAS (strain SCY4-pEPNAS). Both contained the *AtNAS2* ORF from *Arabidopsis* under the control of the GAPDH promoter to induce constitutive and strong expression of *AtNAS2*. NA production in the engineered SCY4-pINNAS strain reached up to 328 *μ*g/g wet weight within 48 h when the growth medium was supplemented with glycine and formate (leading to a 100-fold increased accumulation of the usual level of the precursor SAM). The intracellular concentration of NA reached 766 *μ*g/g wet weight within 60 h when the transgene was introduced with the high-copy episomal vector without any noticeable effect on yeast growth (strain SCY4-pEPNAS). As the chemical synthesis of NA for commercial use as an antihypertensive agent is very expensive ($ 350/mg), this makes its production through a relatively simple fermentative process very efficient [22].

## 7. Hormones and Immunological Agents

Engineering microbes for the production of hormones and immunological agents has also been described. Mazor and coworkers have reported facile isolation of full-length immunoglobulin G antibodies from combinatorial libraries expressed in *E. coli *[51]. Full-length heavy and light chains are secreted into the periplasmic space, where they assemble into aglycosylated IgGs that are captured by an Fc-binding protein anchored in the inner membrane of the bacterium.

The use of *Bacillus subtilis* for recombinant protein expression has been well established. Advantageously, this bacterium is able to secrete functional extracellular proteins directly into the culture medium, making their purification easy; it is not pathogenic for humans and lacks endotoxins in the cell wall. *B. subtilis* was used for establishing a system for the suitable production of human interleukin-3 (hIL-3), which is a cytokine that regulates blood-cell production by controlling the production, differentiation, and function of granulocytes and macrophages [52]. To facilitate optimal secretion of hIL-3 by *B. subtilis*, three signal peptides were tested: the modified AmyL, Pel, and SacB signal peptides. Use of the modified AmyL signal peptide resulted in a reproducibly high secretion from the bacterial cell. The other signal peptides used (SacB and Pel, resp.) did not result in productive secretion of hIL-3 or led to plasmid instability, such that it is impossible to predict which signal is optimal for the secretion of a particular heterologous protein such as hIL-3. The hIL-3 purified showed full biological activity, that is, it was able to specifically induce proliferation of the hIL-3-dependent leukemia cell line MO7e. The best production system was reported for the *B. subtilis* WB800 strain in combination with the pP43LatIL3 vector containing the AmyL signal peptide, yielding 100 mg/L hIL-3 within 24 h of culture.

As *B. subtilis*, *E. coli *is a common host used for over-expression of proteins that do not require glycosylation. This is due to the fact that recombinant proteins can easily be transported in and easily released from the periplasmic space by osmotic shock and that there are fewer proteases in the periplasm compared to the cytoplasm. According to this, periplasmic production of human interferon-*γ* (hINF-*γ*) but not of human interleukin-2 (hIL-2) by the Tat (twin arginine translocation) pathway in *E. coli* strain BL21-SI was described [53]. The Tat system was identified as a transport mechanism for a specific group of periplasmic proteins [54]. Expression of recombinant proteins was obtained using the pEMR vector containing the Tat-dependent modified penicillin acylase signal peptide (mSP*pac*) driven by the T7 promoter.

*E. coli* was also engineered for the expression of a recombinant gonadotropin-releasing hormone (GnRH) vaccine that could be used as a vaccine for the control of fertility and hormone-dependent diseases [55]. To this aim, a highly effective expression plasmid pED-GNRH3 was constructed to express GnRH3-hinge-MVP in the form of a fusion protein. The recombinant gene encoding GnRH3-hinge-MVP which contains three repeated GnRH units, a fragment of hinge region, and a T-cell epitope of measles virus protein was cloned into *E. coli*. A crucial point was to insure plasmid stability during the fermentation process affecting the production of the recombinant protein. This was achieved by the optimization of many culture conditions including fermentation temperature and the culture medium. Both the stability of the plasmid and the expression of GnRH3-hinge-MVP are of real concern for the industrial production of the engineered strain.

## 8. Antioxidant and Anticancer Agents

Hydroxystilbenes including resveratrol and its derivatives possess a large spectrum of biological activities in human health as cardioprotective, antitumor, and antioxidant agents. Engineering bacteria or yeast for resveratrol might represent a valuable means for its production in large quantities [8, 56–59]. At the same time, the potential role of resveratrol as an antimicrobial compound acting as an allelochemical or a phytoalexin and protecting plants from fungal attacks [60–62] or increasing their antioxidant capabilities has led to numerous studies concerning molecular engineering of this compound in plants [59, 63–66].

**Figure 5:**
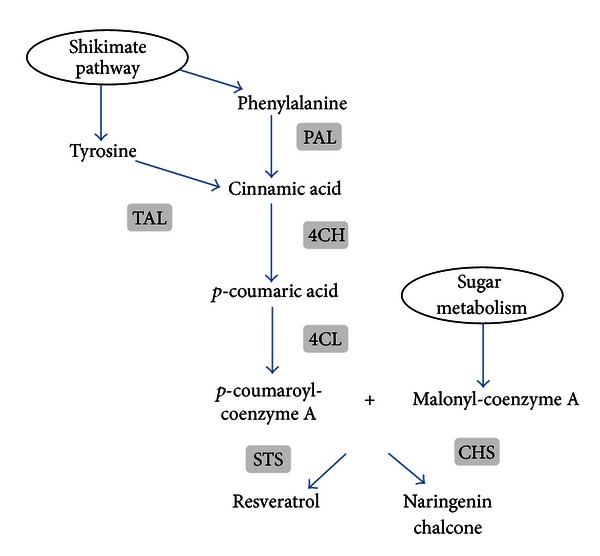
*In vivo* biosynthesis of resveratrol from phenylalanine or tyrosine via the phenylpropanoid/polymalonate pathway. PAL/TAL: phenylalanine/tyrosine ammonia lyase; C4H: cinnamate-4-hydroxylase; C4L: coumarate:coenzyme A ligase; STS: stilbene (resveratrol) synthase; CHS: chalcone synthase.

Resveratrol is obtained through the universal phenylpropanoic acid pathway beginning with phenylalanine (via the phenylalanine ammonia lyase, PAL) or with tyrosine (via the tyrosine ammonia lyase, TAL) (Figure 5). The obtained phenylpropanoic acids are then activated by ligation to coenzyme A by coumarate:CoA ligase (CL), often acting only in the presence of a 4-hydroxyphenyl group and thus termed 4-coumaroyl:CoA ligase (4CL). Hydroxystilbenes are then synthesized by type III polyketide synthases, stilbene synthases (STS) which compete with chalcone synthases, implied in the biosynthesis of the C_6_-C_3_-C_6_ flavonoid ring [67, 68]. PKS act to condense successive units of malonyl-CoA with *p*-coumaroyl-CoA, forming a linear polyketide molecule before cyclization. 

Two main strategies have already been used in the engineering of resveratrol in bacteria or yeast: (i) introducing the entire biosynthetic pathway using aromatic amino acids as substrates (L-phenylalanine or L-tyrosine), and (ii) introducing specific genes, such as *4CL *and* STS*, starting with *para*-coumaric acid as a substrate.

Engineering the entire pathway consists in the production of resveratrol from its precursor phenylalanine or tyrosine through the introduction of the genes encoding for PAL or TAL, the cinnamate-4-hydroxylase, leading from cinnamate to *p*-coumarate (4CH), 4CL, and STS activities. The biosynthetic pathway has been introduced successfully into the oleaginous yeast *Yarrowia lipolytica* completed by constitutive expression of malonyl-CoA, and resulting in a resveratrol production of 1.46 mg/L [69]. The entire resveratrol pathway has been introduced in other microorganisms such as the baker yeast *S. cerevisiae*, molds such as *Aspergillus niger* and *A. oryzae,* and bacteria such as *Lactococcus lactis* and *E. coli*. In all cases, pathway expression started with PAL except for *E. coli* in which TAL was the first enzyme introduced [70]. Resveratrol synthesis was only reported in *E. coli* that expressed the *TAL* gene upon feeding with *p*-coumarate. The necessary addition of *p*-coumarate to the culture medium has the consequence that the TAL or PAL activities remained low in the bacterium. As a result, much of the ongoing efforts are dedicated to increasing these activities in bacteria, for example, by aiming to develop novel TAL or PAL enzymes [71, 72]. Two more recent works constructed the complete resveratrol biosynthetic pathway in *S. cerevisiae* to produce resveratrol from phenylalanine. Trantas and coworkers reported a resveratrol production of 0.29 mg/L within 120 h of cultivation after feeding with 10 mM of phenylalanine [73]. Finally, Shin and coworkers engineered the *S. cerevisiae* strain (W303-1A) with four heterologous genes, the *PAL* gene from *Rhodosporidium toruloides*, the *C4H* and the *4CL1* genes from *A. thaliana,* and the *STS* gene from *Arachis hypogea *[74]. Overexpression of the acetyl-CoA carboxylase (*ACC1*) gene for increasing the pool of malonyl-CoA resulted in an engineered strain termed W303-1A/ACC1 harboring the p425PAL, p423C4H, and pESCTRP4CL1-STS plasmids capable of synthesizing 5.8 mg/L resveratrol upon feeding with 12 mM tyrosine in YP medium containing 2% galactose.

Introducing selective genes is an alternative strategy directed towards transforming microorganisms with only two genes (*4CL* and *STS*) under utilization of *p*-coumaric acid. In fact, *p*-coumaric acid can be used advantageously as a precursor for the bioproduction of resveratrol in engineered bacteria rather than the basic amino-acid phenylalanine. This amino-acid has indeed to be converted to *p*-coumaric acid from cinnamic acid via the P450 enzyme, C4H. However, P450 monooxygenases failed to be effectively expressed in *E. coli.* Additionally, *p*-coumarate is a relatively cheap precursor useful for bioproduction systems [75].

Recombinant *E. coli* strains able to produce resveratrol were obtained [76–78]. An accumulation of 3.6 mg/L resveratrol was reported in the *E. coli* BL21 (DE3) engineered strain through an increase in malonate transport and malonyl-CoA synthase activity [78]. Transformation of the BL21 *E. coli* strain with the *4CL* gene from tobacco and the grapevine *STS* gene resulted in a resveratrol production of 16 mg/L [76]. Similarly, a metabolically engineered *E. coli* strain, which was transformed with the *4CL* gene from *A. thaliana* and a *STS* gene from *A. hypogea*, resulted in a final high titer >100 mg/L of resveratrol [77]. Replacement of the *4CL* gene from *A. thaliana* by the *4CL* gene from *Lithospermum erythrorhizon* in the same JM109 *E. coli* strain, leads to a resveratrol production of 171 mg/L after feeding with *p*-coumaroyl-CoA [79]. When modified cinnamoyl-CoA was used, unnatural stilbenes were formed, suggesting that engineering microbes paves the way for the biosynthesis of novel compounds with potent biological activities [79].

An original approach was developed more recently by the group of Lim and coworkers in which two different *E. coli* strains (BL21 Star and BW27784), two different promoters (the strong T7 promoter and the constitutive promoter of the glyceraldehyde-3-phosphate dehydrogenase), and a library of two 4CLs from *Petroselinum crispum* and *A. thaliana* and nine STS (from pine, *Polygonum*, *Psilotum*, *Vitis,* and *Arachis*) in different combinations were systematically examined to optimize resveratrol production from *p*-coumaric acid in *E. coli *[75]. Both STS from *Vitis vinifera* (*VvSTS*) gene and from *A. hypogea *(*AhSTS*) gene were more efficient in producing resveratrol when coupled with the 4-CL from *A. thaliana* (*At4CL*) gene than with the 4CL from *Petroselinum crispum* (*Pc4CL*) gene. The best performing system was observed with the BW27784 pUCo-*Vv*STS-*At*4CL strain, at which 0.05 mM cerulenin was added to the culture medium for improving the malonyl-CoA pool. Resveratrol production from *p*-coumaric acid reached the impressive value of 2.3 g/L. Due to the prohibitive cost of cerulenin, the simultaneous overexpression of both the acetyl-CoA carboxylase (*ACC*) gene and the biotin ligase (*BirA*) gene from *Photorhabdus luminescens* was obtained allowing acetyl-CoA to be more efficiently converted to malonyl-CoA. 

There are many reports of selective genes being introduced in *S. cerevisiae*, resulting in various productions of resveratrol ranging from 0.65 to 5.8 mg/L [76, 80, 81]. An engineered yeast strain W303-1A containing a *4CL* gene (*4CL1* gene) from *A. thaliana* and as *STS* gene from *A. hypogea* produced unglycosylated resveratrol at a titer of 3.1 mg/L after feeding with 15.3 mg/L *p*-coumaric acid, that is, 14.4 mol% yield [82]. When the 4CL and the STS-encoding genes added to the yeast genome were submitted to protein fusion, expression of the 4CL::STS fusion protein increased resveratrol production in yeast by up to 10-fold (5.25 mg/L resveratrol). This can be compared to the coexpression of 4CL and STS without any protein fusion (0.65 mg/L) [81]. Works also clearly demonstrated that synthetic scaffolds can optimize engineered metabolic pathways. Use of a synthetic scaffold to recruit the 4CL1 and STS enzymes of the resveratrol pathway improved resveratrol production in *S. cerevisiae*, resulting in a 5-fold improvement of the resveratrol production over the nonscaffolded control (14.4 mg/L) [83]. Finally, engineered *S. cerevisiae* yeast cells expressing *4CL* and *STS* genes along with the *araE* gene encoding for a high-capacity *E. coli* transporter exhibited a resveratrol production of 3.1 mg/L [84].

The efficacy of recombinant microorganisms for resveratrol production depends on various factors, such as the species and the strain, the origin of the transferred genes, and culture conditions as well as other parameters such as plasmids or precursors used. A recent study has underlined the crucial role played by the culture medium [85]. Resveratrol production by an industrial Brazilian sugar cane yeast expressing the *4CL1* gene from *A. thaliana *and the grapevine *STS* gene, in a rich medium, reached up to 391 mg/L.

## 9. Conclusions

 There is an increasing number of examples demonstrating that engineering microbial cells for the production of natural compounds of pharmaceutical interest is a successful method to gain access to some bioactive substances only accumulating in low amounts in plants and whose total chemical synthesis is too complex. Moreover, recovery of unnatural compounds with significant biological properties has also been reported through metabolic engineering of microbes [79].

 Some particularly interesting results have been obtained for engineered systems where relatively simple genetic constructs are needed. Extraordinary progresses have also been done in the engineering of very complex biosynthetic pathways such as benzylisoquinoline alkaloids, artemisinic acids or taxol pathways. Stephanopoulos and co-workers recently achieved the production of taxadiene, a key intermediate in the route for taxol, at low cost in *E. coli *[23]. However, still work has to be done before optimizing taxol production by microorganisms as many steps remain to be engineered until reaching taxol.

 Moreover, successful endeavors in the future for optimizing microbial production of natural compounds will require, besides the functional integration of a complete or a partial heterologous pathway in a given microbe, adaptation of the host organism to its environment to improve product titers. For example, resveratrol production by a recombinant industrial yeast strain has been shown to be considerably enhanced according to the culture medium used [85].

New techniques for metabolic engineering will be exploited in the next years to come. Namely, methodologies for generating high-quality libraries of enzyme variants [86] and novel high-throughput screening (HTS) technologies [87] will open the way for the engineering of enzymes for the biosynthesis of various compounds with potent biological activities. Specifically, HTS technologies can rapidly lead to the identification of genes which modulate a particular biosynthesis pathway. Gathering all genes encoding for a biomolecular pathway will allow the assembly of genetic constructs for the synthesis of a given product. New methods for the rapid cloning of single genes together with the availability of synthetic operons, such as bacterial operons (commonly used in the biosynthesis of many medically and pharmaceutically important compounds), have accelerated the construction of synthetic multigene pathways [88, 89]. A very recent application was the coordinated induction of a five-gene pathway leading to zeaxanthin production in tailored *S. cerevisiae* [90].

A major challenge in metabolic engineering is to achieve an optimal flow through a given heterologous metabolic pathway for high yield and productivity. A simple method to fine tune metabolic pathways, named customized optimization of metabolic pathways by combinatorial transcriptional engineering (COMPACTER), was described for rapid tuning of gene expression in a heterologous pathway under different metabolic backgrounds. This was achieved by the creation of a library of mutant pathways using *de novo* assembly of promoter mutants for each pathway gene in a target microorganism followed by high-throughput screening/selection [91].

Finally, systems biology, metabolic engineering, and “omics” technologies (genomics, functional genomics, and metagenomics) have opened the way for protein and biomolecular pathway engineering. Production of novel small molecules of pharmaceutical interest by designing novel proteins and pathways is also under progress [92]. These new methodologies will thus pave the way for very important advances in metabolic engineering of microbial cell factories.
